# Odontogenic rhinosinusitis and sinonasal complications of dental disease or treatment: prospective validation of a classification and treatment protocol

**DOI:** 10.1007/s00405-018-5220-0

**Published:** 2018-11-27

**Authors:** Alberto Maria Saibene, Fabio Collurà, Carlotta Pipolo, Antonio Mario Bulfamante, Paolo Lozza, Alberto Maccari, Flavio Arnone, Filippo Ghelma, Fabiana Allevi, Federico Biglioli, Matteo Chiapasco, Sara Maria Portaleone, Alberto Scotti, Roberto Borloni, Giovanni Felisati

**Affiliations:** 1 Otolaryngology Unit, Department of Health Sciences, San Paolo Hospital, University of Milan, Via A. di Rudinì, 8, 20142 Milan, Italy; 2 Disabled Advanced Medical Assistance Unit, Department of Health Sciences, San Paolo Hospital, University of Milan, Milan, Italy; 3 Maxillofacial Surgery Unit, Department of Health Sciences, San Paolo Hospital, University of Milan, Milan, Italy; 4 Oral Surgery Unit, Department of Health Sciences, San Paolo Hospital, University of Milan, Milan, Italy

**Keywords:** Sinusitis, Paranasal sinuses disease, Endoscopic sinus surgery, Computed tomography, Dental implants, Sinus lift

## Abstract

**Purpose:**

Odontogenic sinusitis and sinonasal complications of dental disease or treatment (SCDDT) represent a heterogeneous group of conditions that often require multidisciplinary care. The present study aims to prospectively validate a classification and treatment protocol for SCDDT patients.

**Methods:**

One hundred twenty-eight consecutive patients (73 females and 45 males, mean age 52.4 years) affected by SCDDT not responding to dental and medical therapy were classified and surgically treated according to the proposed protocol. The protocol classified patients into three aetiology-based groups (preimplantologic, implantologic, and related to traditional dental diseases and procedures, respectively). The groups were further divided into classes according to the presence of oro-antral communications and/or dislocated dental hardware. Each condition was treated according to the class-related, protocol-defined treatment, by either a transnasal or combined transnasal/transoral approach. All patients were successfully classified according to our protocol. None of the proposed classes were redundant, and no condition fell outside the definitions.

**Results:**

The surgical treatment protocol proved to be adequate and effective, in that 125 of the 128 patients completely recovered after surgical treatment.

**Conclusions:**

The term SCDDT and the consequent classification proposed by the authors appear, therefore, to be nosologically correct. Furthermore, the protocol-related proposed treatment appears to be clinically sound, with a success rate nearing 98%.

## Introduction

In the 2012 European Position Paper on Rhinosinusitis [1], odontogenic rhinosinusitis (OS) is briefly mentioned as a possible cause of chronic rhinosinusitis (CRS), but only a limited description of OS is given. Nevertheless, there is some evidence of an increase of cases; some studies report that the odontogenic aetiology accounts for 10–30% of cases of maxillary rhinosinusitis [2, 3], affecting 75% of patients with symptomatic unilateral maxillary rhinosinusitis undergoing surgical treatment [4].

Despite frequent reports of bilateral involvement [5, 6], OS is usually suspected only in patients with unilateral rhinosinusitis with a longstanding history of maxillary dental problems or a recent history of a maxillary dental procedure. Moreover, odontogenic aetiology is often overlooked as a cause of rhinosinusitis by otolaryngologists, dentists, and radiologists, often due to an inadequate consensus on pathological conditions to consider as being OS [7].

While OS has been traditionally related to dental conditions [8], more recent views [9] have attempted to integrate into the definition of “sinonasal complications of dental disease or treatment” (SCDDT) implant-related and sinus augmentation-related sinonasal conditions [10, 11]. OS and SCDDT usually occur when Schneider’s membrane integrity is compromised by dental pathologies or iatrogenic causes [8, 12–14]. Due to the interruption of the mucoperiosteum, there is a higher chance of infections caused by microorganisms, which often include anaerobes and occasionally unusual oral species that ascend from the oral cavity [15–17].

Given the different aetiology and the frequent findings of retained dental metal hardware or foreign bodies, the management of these patients requires a surgeon with a proper training both in the area of endoscopic sinus surgery (ESS) and in that of oral surgery [18, 19]. Although ear, nose, and throat (ENT) specialists or oral/maxillofacial surgeons may possess all the required skills, a multidisciplinary cooperation between ENT and oral/maxillofacial surgeons is advised to provide the best possible treatment.

The management of SCDDT poses, therefore, a significant challenge, both from a nosological and a clinical point of view. In this regard, our group proposed in 2013 a treatment protocol based on the aetiology of the sinonasal involvement (a preimplantologic treatment, an implantologic treatment, or a classic dental treatment or condition) (Table 1) [9]. The aim of the present work was to prospectively validate the protocol for patients suffering from SCDDT who underwent ESS following the failure of antibiotic and focal dental treatment.

**Table 1:** Classification and surgical protocol

Group	C	Condition	Treatment	Cases
I (pre-implantological treatment complications)	1	Sinusitis following maxillary sinus lift with OAC	Combined: FESS + infected material removal + OAC repair	14
II (implantological treatment complications)	2a	Peri-implant osteitis with sinusitis/subperiosteal implant with sinusitis	Combined: FESS + implant removal + OAC repair	13
	2b	Implant dislocation with sinusitis and OAC	Combined: FESS + implant removal + OAC repair	3
	2c	Implant dislocation with sinusitis	Implant removal + FESS	6
	2d	Implant dislocation without sinusitis	Canine fossa approach/transnasal endoscopy	3
III (“classic” dental disease and treatment complications)	3a	Bacterial or fungal sinusitis with OAC	Combined: FESS + OAC repair	31
	3b	Bacterial or fungal sinusitis	FESS	58

The table shows the surgical treatment protocol according to type of complication and presents the patient numerosity in the study accordingly. In case a patient fulfills the criteria for two or more classes, he/she is assigned to the uppermost class shown in the table (which is designed to have on top the most difficult-to-treat scenarios and at the bottom the most easily manageable conditions, thus defining a classification priority)

*G* group, *C* class, *FESS* functional endoscopic sinus surgery, *OAC repair* oro-antral communication repair

## Materials and methods

This study was approved by the Institutional Review Board of the San Paolo Hospital, and a written informed consent was obtained from all patients prior to inclusion.

The study included 128 consecutive patients (73 females and 45 males, mean age 52.4 years) who had been diagnosed with SCDDT not responding to dental and medical therapy and who underwent ENT evaluation between January 2013 and August 2016 in two tertiary care centres (i.e., Department of Otolaryngology, San Paolo Hospital, Milan, Italy and Unit of Maxillofacial Surgery, Istituto Stomatologico Italiano, Milan, Italy).

The inclusion criteria were as follows: (1) diagnosis of SCDDT not responding to adequate focal dental treatment (either surgical or endodontic) and medical treatment; (2) the existence of ENT specialist and dentist/maxillofacial surgeon agreement on the odontogenic focus; and (3) the availability of a presurgical maxillofacial computed tomography (CT) scan (either with or without contrast medium). Exclusion criteria were (1) history of CRS (with or without polyps) replacing dental treatment(s) and (2) sinonasal cavity malignancy [5].

All patients were classified and surgically treated in accordance with the protocol published by our group, which is based on the aetiology and extent of the disease [9].

The classification and treatment protocol are reported in Table 1. Our classification is based on the following three groups: group I, preimplantological treatment; group II, implantological treatment; and group III, dental treatment. Groups are further divided into classes according to the presence of oro-antral communication (OAC) and/or retained metal hardware (Table 1). When a patient fulfilled the criteria for two or more classes (e.g., a complication following maxillary sinus augmentation with peri-implant osteitis), the patient was assigned to the highest class shown in Table 1 (which is designed to have on top the most difficult-to-treat scenarios and, at the bottom, the most easily manageable conditions, thus defining a classification priority).

Prior to surgery, all patients underwent a combined ENT and oral/maxillofacial evaluation to evaluate them for signs and symptoms of rhinosinusitis through nasal endoscopy and oral examination. All patients underwent a cone beam CT head scan for final diagnosis and surgical planning. Orthopantomography and intraoral radiography scans were ordered in selected cases according to the dental/maxillofacial surgeon’s needs.

All patients received pre- and post-surgical antibiotic therapy, based on oral levofloxacin 500 mg q.d., for 5 and 10 days, respectively. Allergic patients were treated both presurgery and postsurgery with oral cefuroxime axetil 500 mg twice daily; we preferred not to use amoxicillin/clavulanate because it is typically ineffective against SCDDT-causing bacteria [20]. The patients were then treated under general anaesthesia with a surgical procedure chosen according to the class-defined surgical protocol (i.e., either exclusively transnasal or combined transoral and transnasal; see Table 1). According to the sinonasal involvement shown by the CT scan, the ESS was composed by one or more of the following procedures: for maxillary involvement with sinusitis, patients underwent total uncinectomy and wide middle antrostomy; for maxillary involvement without sinusitis, patients underwent partial inferior uncinectomy and mini-antrostomy, sized the smallest for allowing for the removal of retained implant hardware; for ethmoidal involvement, patients underwent radical ethmoidectomy; for frontal involvement, patients underwent a type I frontal drainage according to the Draf classification; and, for sphenoidal involvement, patients underwent sphenoidotomy performed either through the sinus natural ostium or, in case of ethmoidal concurrent involvement, using a transethmoidal approach.

All patients performed nasal washes with saline solution and applied nasal niaouli oil for 30 days after surgery between three and four times per day. All patients underwent ENT examination at 7 days, 30 days, and 60 days after surgery; however, in cases of persistent infection, patients were examined weekly and then, following complete recovery, underwent the aforementioned regular ENT examination. Patients with a minimum follow-up of 6 months were included in the present study. Treatment success was considered achieved when the patient reported complete resolution of symptoms and no signs of mucosal inflammation were detected by nasal endoscopy in all the originally involved sinuses (i.e., no pus, crusting, or mucosal swelling). No CT scans were performed after treatment to confirm the endoscopy findings of successful treatment. Treatment success rate as above-defined was evaluated for each group.

## Results

All patients were successfully and classified within one of the protocol classes. No class resulted redundant, and at least three patients were assigned to each class.

The overall treatment success rate (as above-defined; self-reported symptom resolution and endoscopically observed-involved sinus healing) was 97.65%. One patient (class 1) required further antibiotic therapy, another one (class 3b) required a second surgical procedure after antrostomy closure, and a third patient (class 1) was lost to follow-up after disease recurrence. No intraoperative complications such as major bleeding, lamina papyracea, or orbital breaches or cerebrospinal fluid leaks were reported. No anaesthesia-related adverse events were recorded. No patient had perioperative orbital complications, bleeding events, or cerebrospinal fluid leaks. Of the total patient population, 7.81% were dismissed on the same day of surgery, 85.93% were discharged the day after, and the remaining 6.25% were dismissed after two nights in the hospital, respectively.

In regard to patient presentation, the clinical scenario and extent of sinonasal involvement was the following (Table 2). “Classic” dental treatments and conditions (i.e., those not related to implantology or pre-implantologic procedures; group 3) were the most common cause of SCDDT, found in 89 patients. Additionally, 31/89 (34.8%) patients with SCDDT not related to implantological or preimplantological procedures associated with an OAC (class 3a); 5/89 (5.6%) had unilateral maxillary involvement; 25/89 patients (28.1%) had unilateral extramaxillary involvement; and 1/89 (1.1%) had bilateral involvement. Of the study participants, 58/89 (15.7%) patients demonstrated OS without OAC (class 3b), while 14/89 (15.7%) had unilateral maxillary involvement, 37/89 (41.6%) had unilateral extramaxillary involvement, and 7/89 (7.9%) had bilateral involvement. None of these patients had peri-implant osteitis or retained implant hardware. Patients treated for implantologic surgery complication (group 2) were 25 in number; of these, 9/25 (36.0%) had unilateral maxillary involvement and 16/25 (64.0%) had unilateral extramaxillary rhinosinusitis. Additionally, among these 25 patients, 13/25 (52%) had peri-implant osteitis without implant dislocation (class 2a), 3/25 (12%) had an OAC with implant dislocation (class 2b), 6/25 (24%) had an implant dislocation with sinusitis, and 3/25 (12%) an implant dislocation without sinusitis. Fourteen patients who underwent surgery for preimplantological complication (group 1); 12/14 (85.7%) of them had unilateral extramaxillary involvement, 1/14 (7.1%) had unilateral maxillary involvement, and 1/14 (7.1%) had bilateral involvement. None of these patients had peri-implant osteitis or retained metal implant hardware. Table 2 provides a brief overview of these reported results.

**Table 2:** Patient classification and clinical results

		Unilateral maxillary involvement		Unilateral extramaxillary involvement		Bilateral involvement		Total (*n*)
		*n*	Rate (%)	*n*	Rate (%)	*n*	Rate (%)	
Total SCDDT patients		29	22.7	90	70.3	9	7.0	128
Groups								
I	Preimplantologic surgery complication	1	7.1	12	85.7	1	7.1	14
II	Implantologic surgery complication	9	36.0	16	64.0	0	0.0	25
III	“Classic” dental treatment complication	19	21.3	62	69.7	8	9.0	89
Classes								
1	Sinusitis following preimplantologic surgery	1	7.1	12 (1)	85.7	1	7.1	14
2a	Sinusitis with perimplantitis/subperiosteal implant and OAC	3	23.1	10	76.9	0	0.0	13
2b	Sinusitis following implant dislocation with OAC	1	33.3	2	66.7	0	0.0	3
2c	Sinusitis following implant dislocation	2	33.3	4	66.7	0	0.0	6
2d	Implant dislocation	3	100.0	0	0.0	0	0.0	3
3a	Odontogenic sinusitis with OAC	5	16.1	25 (1)	80.6	1	3.2	31
3b	Odontogenic sinusitis	14	24.1	37 (1)	63.8	7	12.1	58

The table shows the case series composition, according to the SCDDT classification along with the degree of sinonasal involvement. All rates are calculated among homogeneous classes and groups. Numbers in brackets indicate patients who failed after the first surgical treatment

*OAC* oro-antral communication, *SCDDT* sinonasal complications of dental disease or treatment

During the surgical procedure, 54/128 patients were recorded as showing signs of ostio-meatal complex obstruction. Six of them showed signs of inflammatory degeneration of the mucosa of the ethmoid and/or ostio-meatal complex, while the remaining 48 had anatomic anomalies such as septal deviation, massive inferior turbinates hypertrophy, or middle turbinate concha bullosa, which possibly pre-existed the SCDDT.

## Discussion

Often overlooked as a cause of sinonasal disease by otolaryngologists, dentists, and radiologists, OS deserves special consideration because it differs in terms of its microbiology; pathophysiology; and, consequently, management in comparison with “rhinogenic” sinonasal disease [21]. As reported by Albu and Baciut [22], the majority of rhinosinusitis guidelines written in the last decade do not include OS as a cause of CRS. OS is infrequently mentioned in the most recent guidelines and the literature lacks coverage of standard protocols regarding its diagnosis and management [23].

In 2013, our group proposed the new concept of SCDDT and a new classification system and subsequently focused on establishing a treatment protocol [9]. The protocol introduces the need for combining ESS and intraoral approaches for some specific aetiopathogenetic cases, such as graft displacement after sinus augmentation, sinonasal foreign bodies (e.g., implants, teeth, dental tools), and oro-antral communications. This protocol was introduced prospectively in our daily SCDDT treatment practice guidelines. Surgery proved to be adequate and effective, as 97.7% of patients completely recovered, in contrast with other studies where the disease recurred with consequent need for redo surgery in 9–14% of patients [24]. However, one of the weak points of our protocol is the administration of pre- and post-operative antibiotic therapy, which is not supported by guidelines or any other evidence besides our daily practice. Therefore, the choice to perform such antibiotic therapy in our case series might lead to some bias in interpreting our positive results, and this has to be taken into account. The rationale of the antibiotic therapy of choice for this paper lies in our previous case series [9], where we noted that most treatment failures in the patient cohort occurred in those who did not receive quinolone therapy. Further insight into the postoperative therapy of SCDDT patients is definitely required and only the publication of other large case series could enable to production of strong guidelines.

The results obtained by authors who have already introduced our protocol into their daily practice seem to be in line with the findings of our reports. More specifically, Fadda et al. applied the same protocol to 31 patients and all of them demonstrated improvements in rhinosinusitis symptoms as confirmed by clinical examination and CT scan; additionally, no significant complications were recorded and no instance of revision surgery was required [12].

From our standpoint, the need for a different protocol for OS patients comes naturally if we consider that this group of conditions requires unique diagnostic criteria and a treatment regimen that differs from nonodontogenic rhinosinusitis. Surgical planning for OS patients requires the specific evaluation of the aetiology and the extent of sinonasal involvement. Radiological imaging is an important tool for establishing a diagnosis and includes periapical radiography, panoramic radiography, and CT, with the latter being considered the gold standard for diagnosing odontogenic rhinosinusitis [25]. In a series of 55 patients with OS, Wang et al. [26] noticed that only 65% of radiology reports mentioned dental pathology, and 20% of these patients were only diagnosed after a retrospective analysis of the presentation and outcomes. Another study from our group [5] showed that only 40% of patients had a condition limited to the maxillary sinus, while the remaining percentage demonstrated a more extensive sinonasal involvement. For this reason, OS treatment should focus not only on the maxillary sinus but also on all sinonasal cavities involved (i.e., where CT scans show sinus opacation or any kind of foreign body), as already detailed in the “Materials and methods” section, to minimize the number of recurrences. Imaging has also maintained the pivotal role of differentiating rhinosinusitis caused by obstructed drainage pathways and that caused by odontogenetic disease. Failure to identify the dental or obstructive origin of a sinusitis can lead to the misdiagnosis of an SCDDT case as an instance of CRS. As already stated, SCDDT/OS and CRS are different entities in terms of aetiology, pathophysiology, and management [27].

There are indeed some previous studies in existence that attempted to categorize the dental aetiologic factors of rhinosinusitis according to their frequency and importance; however, they mostly showed a lack of internal and external consistency, suggesting that different (and often variable) dental etiologic factors be included and proposing various methods of diagnosing and managing cases of rhinosinusitis that had dental sources [28]. Multiple studies have shown excellent results with ESS and dental surgery, although the ideal sequence of management is not clear [29]. Wang et al. [26] presented a 55 OS patient case series, whose diagnosis and treatment schedule did not appear very clear. Other studies in the literature show favourable results, recommending dental surgery as a core component of management, but they often propose different treatment protocols for the same pathology [30], Astonishingly, in the era where ESS is recognized as the surgical treatment of choice for chronic rhinosinusitis, the classic Caldwell–Luc approach is still suggested by some authors [31] despite its significant morbidity. In the event that an intraoral approach to the sinus (e.g., for removing grafts) is needed, we tend to prefer less invasive approaches, such as the antral retriever [32] or the creation of a bony window pedicled to Schneider’s membrane [33].

Last, but definitely not least, the close collaboration among maxillofacial/oral surgeons, implantologists, and ENT specialists is pivotal to correctly diagnose and treat complex cases of SCDDT. Multidisciplinary treatment allows for the achievement of a rapid recovery and a minimization of the risk of recurrence. This is especially true for group I complications, a condition in which the presence of grafts and the systematic manipulation of the Schneiderian membrane during the augmentation procedure exposes the maxillary sinus to higher risks when compared with other dental procedures [34, 35].

## Conclusion

In 2013, the authors moved away from the concept of OS, by introducing the definition of SCDDT. This change is not merely dictated by a taxonomic need, since prior dental treatments have almost invariably to be considered the primum movens in this type of conditions. The results herein reported show that the protocol we proposed is outcome-oriented, safe, and effective, with a success rate of nearly 98%. In view of this favourable outcome, we hope that diffusion of the protocol might contribute to a better understanding of the key clinical characteristics of SCDDT, improved physician awareness and management choices, and comparison of treatment results among different groups.
